# Preserving Neural Function under Extreme Scaling

**DOI:** 10.1371/journal.pone.0071540

**Published:** 2013-08-19

**Authors:** Hermann Cuntz, Friedrich Forstner, Bettina Schnell, Georg Ammer, Shamprasad Varija Raghu, Alexander Borst

**Affiliations:** 1 Department of Systems and Computational Neurobiology, Max Planck Institute of Neurobiology, Martinsried, Germany; 2 Institute of Clinical Neuroanatomy, Goethe University, Frankfurt/Main, Germany; 3 Ernst Strüngmann Institute for Neuroscience in Cooperation with Max Planck Society, Frankfurt/Main, Germany; 4 Department of Biology, University of Washington, Seattle, Washington, United States of America; 5 Neuroscience Research Partnership, Biopolis, Singapore; McGill University, Canada

## Abstract

Important brain functions need to be conserved throughout organisms of extremely varying sizes. Here we study the scaling properties of an essential component of computation in the brain: the single neuron. We compare morphology and signal propagation of a uniquely identifiable interneuron, the HS cell, in the blowfly (*Calliphora*) with its exact counterpart in the fruit fly (*Drosophila*) which is about four times smaller in each dimension. Anatomical features of the HS cell scale isometrically and minimise wiring costs but, by themselves, do not scale to preserve the electrotonic behaviour. However, the membrane properties are set to conserve dendritic as well as axonal delays and attenuation as well as dendritic integration of visual information. In conclusion, the electrotonic structure of a neuron, the HS cell in this case, is surprisingly stable over a wide range of morphological scales.

## Introduction

Intuition from simple cable theory tells us that smaller neurons should have larger input resistances, faster integration times and be altogether electrically more compact (e.g. [1]). However, the brains of smaller organisms which consist of correspondingly smaller cells (e.g. [2]–[6]) often implement very much the same computations and functions as their larger counterparts. Since brain tissue is energetically expensive to maintain [7]–[9] the question arises why brain evolution did not lead to more compact brains even in larger organisms? How does the single neuron cope with the electrotonic changes due to differences in size? Would a neuron compromise material costs that are known to be otherwise instrumental in determining dendrite structure [10]–[12] to adjust its shape to preserve a particular computation? To address these questions the concept of conservative scaling may be useful (e.g. [3], [13]). In such a setting an invariance preserving important electrotonic properties rather than anatomical proportions could result in a conservation of dendritic integration features such as relative conduction delays and non-linear interactions of synaptic currents in the dendrite.

**Figure 1:**
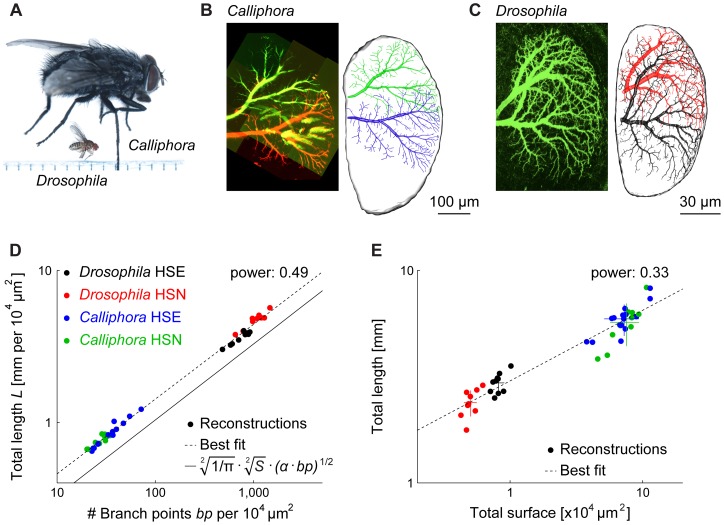
Morphological analysis of *Drosophila* vs. *Calliphora* HS cell dendrites. (A) Comparison of size between the blowfly (*Calliphora*) and the fruit fly (*Drosophila*); ruler has mm markings. (B) Superposition of the HSN (green) and the HSE (red) in a Lobula Plate of *Calliphora*. On the right side, a rendering of the full reconstructions of both cells (HSN – green and HSE – blue) within the marked boundaries of the reconstructed Lobula Plate is depicted. (C) Similar setting for the HSN and HSE cells (both are green since they both express GFP) in a brain of *Drosophila* with corresponding reconstructions (HSN – red and HSE – black) to the right. (D) Power law between branch point and total length densities, a power of 1/2 being indicative of optimal wiring for planar dendrites [33]. (E) Absolute scaling between total surface and total length. Crosses indicate population mean and standard deviation.

Here, we address these questions in a circuit of the fly visual system for which both the function and the underlying biophysical mechanisms are well understood at the cellular and the network level: Tangential Cells (LPTCs) of the third visual neuropil, the Lobula Plate, form a circuitry involved in optic flow calculations. By pooling of inputs from presynaptic elementary motion detectors and cross-talk between LPTCs they compute large field visual motion features required for the fly's course control [14]. In a number of electrophysiology and modelling studies on *Calliphora* LPTCs, their predominantly passive electrotonic features [15]–[17] were characterised and linked to their function as large-field signal integrators: They were shown to average out spatial structure in the motion image [18], to communicate signal features selectively to other LPTCs (e.g. [19], [20]) and to compartmentalise the signals between their dendrites and axons [21]–[23]. More recently, LPTCs have also become amenable to intracellular electrophysiological analysis in *Drosophila* [24], [25] revealing surprisingly conserved functionality and visual responses. This opens up the opportunity to compare both electrophysiology and shape of an identified neuron with its exact homolog in two flies of fundamentally different sizes (Figure 1A), with a scaling factor of about four in each dimension. LPTCs are the ideal subject to study the scaling property of one particular neuron since each LPTC is individually identifiable due to the high degree of constancy in receptive field, morphology, location within the Lobula Plate and visual response properties [26], [27].

In this study we focus particularly on a subset of LPTCs, the Horizontal System (HS) cells, which respond selectively to horizontal large-field motion. Their membrane potential responds in a graded direction-selective manner, i.e. it depolarises during front-to-back visual motion stimulation and hyperpolarises when stimulated in the opposite direction. Three individual HS cells exist in each of the two optic lobes of the fly brain. They are named according to their position within the Lobula Plate, with HSN (Northern) covering the dorsal, HSE (Equatorial) the intermediate and HSS (Southern) the ventral parts, respectively [26] (Figures 1B and C show the HSN and HSE in *Calliphora* and *Drosophila*, respectively). In the following, we use electrophysiological and morphological data in combination with computational models to quantitatively assess the scaling principles of HS cells in both species.

## Materials and Methods

### Reconstructions and anatomy

*Calliphora* cells were filled intracellularly using sharp electrodes with Alexa 488 [28] for the three dimensional reconstructions. In *Drosophila*, a Gal4 driver (NP0282) driving expression specifically in HSN and HSE in both lobes was used (Figure 1C) [25], [29]. Reconstructions of HS dendrites (see overview in Figure S1) were done using custom-made software in Matlab (The Mathworks, Inc.) and exported to our software package that is freely available for download (the TREES toolbox, www.treestoolbox.org) [10], [30]. Reconstructions are available on the TREES toolbox website and at www.neuro.mpg.de/30330/borst_modelfly_downloads. All further analyses and models were performed using these tools. Reconstruction of the axons was not possible in *Drosophila* HS cells due to co-localisation of other labelled cells in the NP0282 driver line. One axonal reconstruction was obtained using intracellular injection of a fluorescent dye (see below) and was appended to all *Drosophila* dendrites for electrotonic analysis. Combined spanning fields of HSE and HSN cells provided good context clues for the Lobula Plate contours (confirmed with background stains).

### *Drosophila* electrotonic analysis

Whole cell patch-clamp recordings were performed as described previously [24], [25]. Briefly, flies were anaesthetized on ice and waxed on a Plexiglas holder. A small window was cut into the cuticle on the backside of the head and a glass electrode filled with collagenase (Collagenase IV, Gibco, 0.5 mg/ml in extracellular saline) was used to weaken the perineural sheath and expose the somata of LPTCs. Somata were approached with a patch electrode (7–10 MΩ resistance, thin wall) filled with intracellular solution (as in [31] containing an additional 30 mM Alexa Fluor 568 hydrazide-Na (A-10441, Molecular Probes) adjusted to pH 7.3). Signals were recorded on a BA-1S Bridge Amplifier (npi electronics, Tamm, Germany), low-pass filtered at 3 kHz, and digitised at 10 kHz via a D/A converter (PCI-DAS6025, Measurement Computing, Norton, MA) with Matlab. Note that electrophysiological and morphological data were not obtained from the same individuals. Input resistance and membrane time constant were measured in responses to 3× step currents of hyperpolarizing 50 pA each, 30 seconds and 10 minutes after break-in. Membrane time constants were obtained by linear regression on a semi-logarithmic plot corresponding to a single exponential fit to the voltage response, which yielded good results. Both input resistance and membrane time constant increased during the recording period from 176±46 to 205±45 MΩ and from 4.3 ± 1.4 to 4.9±1.3 ms respectively within 10 minutes (numbers are mean and standard deviation). While the quality of the seal increases with time, the quality of the recording decreases because of clogging of the electrode. It was therefore not clear which values to use but the differences were small in comparison to the overall variance in experimental values. The later measurements were used for averaged values. Since recordings were obtained in current clamp and the membrane potential measurements were relatively noisy even without any stimulation, we were unable to accurately estimate and compensate for the series resistance. However, our values for the input resistance are in good agreement with data for VS cells in *Drosophila* obtained in voltage clamp [32], suggesting that errors in measurement are minor.

### Morphological model

To check that *Drosophila* HS cells obeyed optimal wiring constraints we first verified the scaling properties predicted by these constraints (Figure 1D) [33]. Further we performed the complete analysis as previously for *Calliphora* HS cells [28] involving the generation of a morphological model based on optimal wiring principles (see Results). Briefly, dendrite spanning fields were obtained for each reconstructed HS dendrite delimiting the area covered by the dendrite such that each point in the dendrite spanning field is within a threshold distance away from the dendritic tree. Target points were then distributed randomly within the spanning fields and connected to dendritic structures satisfying two wiring costs: (1) the total dendrite length should be short and (2) the length of all paths along the dendrite from any point to the root should be short. The second cost was weighted with a balancing factor *bf* against the first cost. We verified that our morphological model was useful also for *Drosophila* dendrites using a similar model parameter value *bf* as for *Calliphora* HS dendrites. Only few minor adjustments were required in the modelling procedure accounting for the differences in scale: e.g. a finer resolution was used to estimate the dendrite spanning fields.

### Morphological model database

The corresponding database of synthetic dendrites used to studying the electrotonic scaling properties of HS cells were obtained using target point numbers ranging from 625 to 2,300 and scaling down the surface area of a *Calliphora* HS dendrite between 1× and 4.5×. A model for diameter tapering was obtained as discussed previously [12] based on requirements for synaptic democracy. Two sets of parameters (consisting of a terminal branch diameter value and a scaling factor) were obtained by fitting the data from real reconstructions for *Drosophila* and *Calliphora* HS cell dendrites, respectively. All algorithms are available in the Matlab software package (the TREES toolbox, www.treestoolbox.org) [10], [30].

## Results

### Morphological analysis

First, we studied the global scaling properties between the two species of flies. Selected size parameters (Table 1) scaled linearly with a factor of about 4 indicating that isometric rather than allometric scaling takes place regarding the body shape thereby conserving the general anatomical proportions [34], [35]. In particular, the area of the Lobula Plate (see outlines in Figure 1BC) that is spanned by the HS cells seemed to be scaled linearly with the body length and its shape was well preserved. It is worth noting that HSN dendritic fields cover a similar percentage of the Lobula Plate in *Calliphora* and *Drosophila*, whereas the HSE dendritic field covers a larger percentage in *Drosophila* than in *Calliphora*. We have previously shown that dendrite morphology of LPTCs in fact depends most strongly on the area they span [28]. This indicates that some aspects of LPTC morphology should be conserved and should scale linearly with the Lobula Plate size. To make quantitative assertions, however, full morphological LPTC reconstructions were required consisting of connected cylinders representing the tree structure. Such reconstructions were obtained and discussed previously for the *Calliphora* HS cells [28] (Figure 1B). To quantitatively assess differences in morphology and signal propagation between HS cells of *Calliphora* with those of *Drosophila*, we acquired the corresponding data from fruit flies. Studying *Drosophila* allows the usage of genetic techniques. We therefore obtained image stacks from flies using the Gal4 driver line NP0282 to express GFP bilaterally in HSN and HSE cells [25], [29] (Figure 1C). The dendrite reconstructions were obtained from these image stacks using custom-made Matlab code as done previously for the *Calliphora* HS cells (see Methods and Figure S1).

**Table 1:** Scaling of global anatomical features.

Size parameters	*Calliphora*	*Drosophila*	scale	linear scale
body length (mm)	11	2.6	4.2	4.2
brain volume (mm^3^)	1.22	0.015	81.3	4.3
Lobula Plate area (mm^2^)	0.18	0.009	20.0	4.5

*(personal communication, Christoph Kapfer).*

A major determinant of dendritic shape is a strive for minimising wiring costs and conduction times [10]–[12], [36]–[38]. It was previously shown that optimal wiring constraints account for inner branching features in the case of *Calliphora* LPTCs [12], [28]. Assuming optimal wiring principles, the scaling behaviour of dendrites can be predicted in terms of dendrite length, number of synapses, number of branch points and the surface or volume that a dendrite spans [33]. A 1/2 power between branch point density and dendrite cable density is expected for planar dendrites with a precise calculation of a tight lower bound for the optimal dendritic length (Figure 1D, straight line). Both *Drosophila* and *Calliphora* HS cell dendrites were strictly constrained by this equation with the best fit of 0.49 for the power relation between cable density and branch point density (Figure 1D). As expected, the overall density of dendrites was much larger in the smaller *Drosophila* dendrites. Beyond this relation describing the scaling behaviour in terms of optimal wiring it is useful to compare the absolute dendritic length with the surface covered by the dendrite (Figure 1E). For this relation no prediction in terms of optimal wiring is known. A linear relation (power of 1) would indicate that the cable density is similar in both species, while a power of 1/2 would correspond to a simple isometric scaling without a change in dendrite complexity. Interestingly, the fitted power was 0.33 indicating that the larger dendrites of *Calliphora* HS cells were consistently less complex than their smaller counterparts. This result is particularly counterintuitive since *Drosophila* has a much smaller number of facets in the eye with 700 in *Drosophila* [39] vs. 4,500 in *Calliphora* [40]. Since the underlying neural circuitry is subdivided and organised into retinotopic cartridges corresponding to the ommatidial layout [41], *Drosophila* HS cells should in fact receive fewer inputs to be integrated within their receptive field.

### A morphological model for scaling

**Figure 2:**
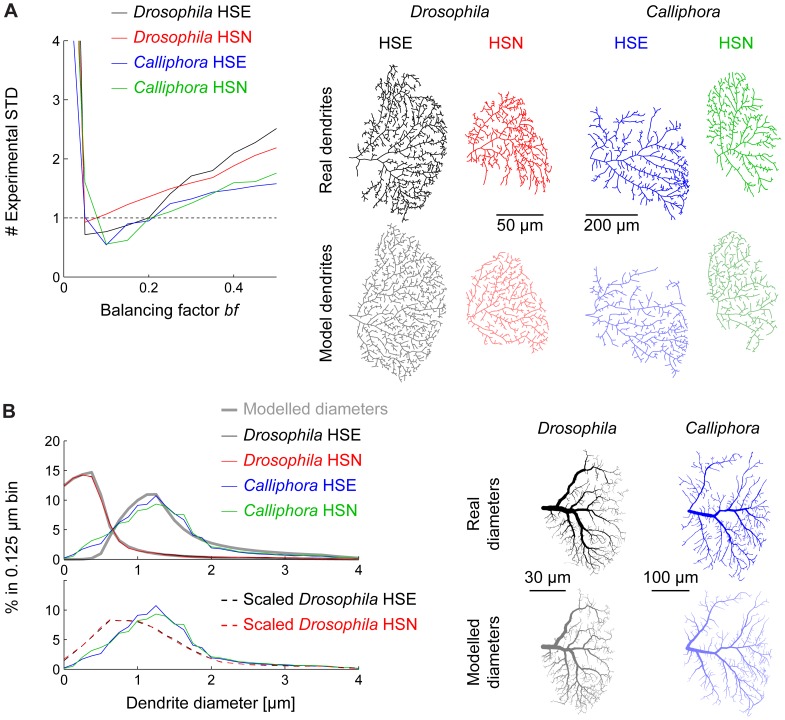
Morphological model to study the scaling properties of HS cells. (A) Model error compared to standard deviation of experimental measures as a function of the balancing factor *bf*, the one parameter in the morphological model. To the right, sample model dendrites (lighter colours) with their respective real counterparts for *Drosophila* HSE (black) and HSN (red) and *Calliphora* HSE (blue) and HSN (green). (B) Diameter histograms for all cell types (same colours as in A) and best fits (grey lines) using the quadratic taper fit from our model [12]. Lower panel shows scaled diameters of *Drosophila* cells in comparison to *Calliphora* cells. To the right, sample *Calliphora* and *Drosophila* HSE dendrites comparing real and modelled diameters (in lighter colours).

In order to understand the change of morphological and electrotonic properties due to scaling we first developed a model that describes the morphology with a few parameters. With the possibility to scale continuously between *Drosophila* and *Calliphora* dendrites the consequences of morphological scaling can then be studied independently from each other while keeping the other features constant. We have recently proposed a morphological model capable of generating synthetic dendrites that match well those of *Calliphora* LPTCs and many other neurons [10], [12], [28]. The model is based on the assumption that a dendrite strives to connect optimally to its inputs that are distributed in space. In the case of LPTCs, inputs are retinotopically organised elementary motion detectors covering the area of the Lobula Plate. Target points that are distributed within the contours of a real LPTC are connected while minimising cable length and path lengths along the tree toward the root [28]. A cost for long path lengths is weighted in comparison to the cost of cable length by one parameter of the model, the balancing factor *bf.* The same procedure was previously performed on *Calliphora* dendrites [28] (see Methods). When branching features (total length, branch order and path length distributions) of the resulting model dendrites were linearly combined and compared to the standard deviation in the experimental measures, a small parameter value *bf*  = 0.1 represented a good fit in *Drosophila* as well as in *Calliphora* (Figure 2A, see also sample dendrites and their corresponding model counterparts). The comparably low value for *bf* (the typical range is between 0.1 in LPTCs and 0.85 in dentate gyrus granule cells for example) seems to discard fast conduction times in favour of short cable length and reduces the effective electrotonic compartmentalisation in favour of more even integration of signals throughout the dendrite [10].

Next, we studied the scaling property of diameters while further confining our model. Beyond the cable length and dendrite complexity, cable diameters play an important role for conveying electrical signals. While average dendrite diameter values (see Table 2) scaled isometrically with the rest of the global fly measures, diameter distributions were slightly different (Figure 2B, top and bottom panel). When dendrite diameters were scaled to have the overall same average diameter, a higher proportion of thin *Drosophila* dendrites was revealed compared to a higher proportion of medium size diameters for *Calliphora* counterparts. A quadratic diameter taper was previously shown to optimise synaptic current transfer democracy in LPTCs [12] and a method exists for mapping diameters onto a tree structure following the corresponding rules of diameter tapering. Beyond reproducing the diameter taper observed in *Calliphora* LPTCs the method generates good diameter mappings for a number of other dendrites [10], [30]. The quadratic diameter taper is parameterised with a parameter for the smallest dendrite tip diameter and a scaling factor determining a neuron's overall leak [12]. To compare diameter values between *Calliphora* and *Drosophila* dendrites we obtained the best fits for these two parameters in the two populations of dendrites. Parameter sets reproducing the diameter distributions were obtained and validated (Figure 2B). This procedure allows us to manipulate diameter values of the morphology using the two different diameter mapping methods as well as a smooth transition between the two. In summary, the morphology of both types of HS cells are essentially scaled versions of each other following similar branching principles but *Drosophila* HS cells are surprisingly more complex than *Calliphora* HS cells.

**Table 2:** Scaling of dendritic anatomical features.

Size parameters	*Calliphora*	*Drosophila*	scale	linear scale
avg. dendrite diameter (µm)	1.92±0.27 (N = 25)	0.58±0.08 (N = 20)	3.4	3.4

### Designing the passive electrotonic model

**Figure 3:**
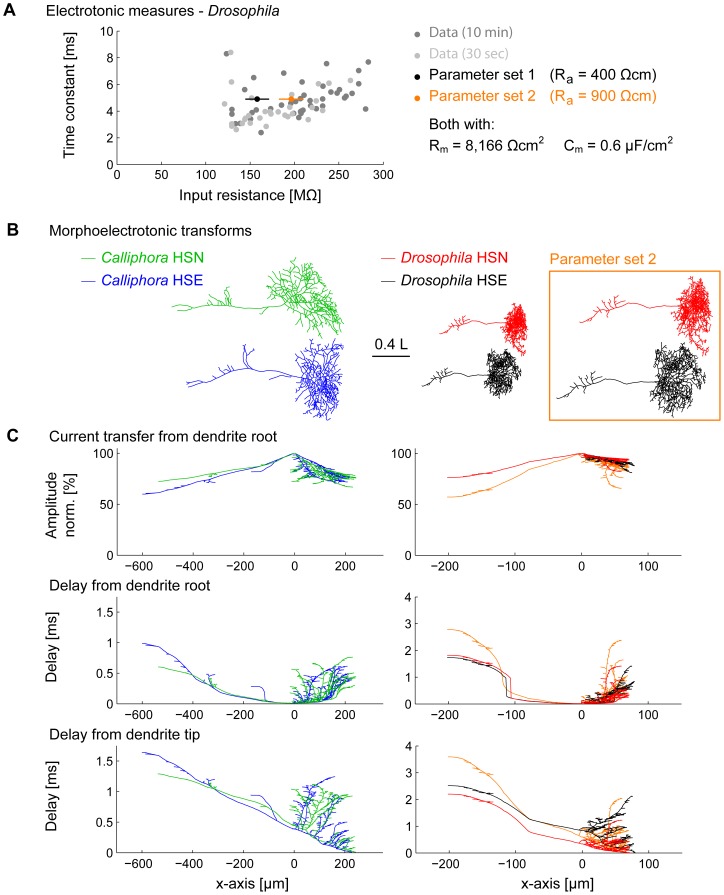
Signal conduction and dendritic integration in *Drosophila* and *Calliphora* HS cells. (A) Experimental input resistance and membrane time constant measurements in *Drosophila* HS cells (dark grey −10 min.; light grey −30 sec. after breaking into the cell). The later measurements were used for estimating average values since the patch is more stable then. Two model parameter sets (black and orange) were used in the further study. (B) Morphoelectrotonic transforms of four sample cells where electrotonic lengths are mapped onto the segments of the branched structures [42] (orange box: second parameter set for *Drosophila*). (C) Same four cells as in B but with the amplitude decay from the dendrite root mapped onto the y-axis of the cells (top panels) and delays from the dendrite root (middle panels) and from selected dendrite tips (bottom panels). The *Drosophila* HSN cell results are shown for the second parameter set in orange.

Next we studied the electrotonic properties of HS cells to determine the following parameters for the corresponding compartmental models: the specific membrane resistance *R_m_*, the specific axial resistance *R_a_*, and the specific membrane capacitance *C_m_*. We determined input resistance and membrane time constant in electrophysiological intracellular recordings (Table 3; see Methods). The measured membrane time constants were short in both species, but about 2.3× longer in *Drosophila* (4.9 ms) than in *Calliphora* (2.1 ms). The measured input resistances were much higher (∼50×) in *Drosophila* HS cells (205±45 MΩ instead of 4–5 MΩ in *Calliphora*). A common assumption is that the specific membrane capacitance is close to *C_m_* = 1µF/cm^2^. The specific membrane resistance *R_m_* is then fully determined when the measured membrane time constant is known. This is the case since *τ*  =  *R_m_* · *C_m_* corresponding to the membrane time constant for a current injection in an infinite cable is valid for current injections in complex electrotonic models of neurons including the ones tested here. For *Calliphora* HS cells, a model was selected that corresponding to the measured membrane time constant of 2.1 ms had *R_m_* = 2,100 Ωcm^2^ and to fit the input resistance *R_in_* required *R_a_* = 100 Ωcm. This is in agreement with previously measured parameters [17]. The *Drosophila* HS cell electrotonic model has not yet been studied and we performed meticulous intracellular recordings for which experimental *R_in_* and *τ* are plotted in Figure 3A. With *Calliphora* HS cell membrane parameters, *Drosophila* HS cells exhibit an input resistance of about 40 MΩ. To obtain realistic input resistance values in the model, *C_m_* was required to be very small and *R_a_* very large even considering that recordings in *Drosophila* were performed in the soma whereas *Calliphora* input resistances measures were performed in the axon. We considered two model parameter sets both with *R_m_* = 8,166 Ωcm^2^ and *C_m_* = 0.6 µF/cm^2^ but with different axial resistances of *R_a_* = 400 Ωcm in a model with realistic axial resistance but with low input resistance and with *R_a_* = 900 Ωcm in a model with very high axial resistance but corresponding to the average experimental input resistance (Figure 3A black and orange dots). *C_m_* = 0.6 µF/cm^2^ and *R_a_* = 400 Ωcm are at the boundaries of typically observed values in invertebrates and therefore within the realistic range (see summarising table 4 in [17].

**Table 3:** Electrophysiological measures.

	*Calliphora*	*Drosophila*
Input resistance, *R_in_* (MΩ)	4.95±2.25 (N = 5)	205±45 (N = 14)
Membrane time constant, *T* (ms)	2.1 (N = 5)	4.9±1.3 (N = 14)

*Data for Calliphora are from* [17].

### Dendritic integration in the electrotonic models

Since the primary computation in LPTC dendrites is the integration of local motion information, dendritic integration properties of *Calliphora* and *Drosophila* electrotonic HS cell models might reflect the similarity in function. Figure 3B shows morphoelectrotonic transforms [42] of four representative morphologies, one for each HS cell type. Instead of showing metric length relations for the individual segments of the branched structures, this representation maps electrotonic length onto the respective segments. Strikingly, in this representation, HS cells of *Drosophila* exhibit very similar proportions and overall size as HS cells of *Calliphora*. The summed electrotonic lengths were remarkably similar (*Calliphora*: 23.6±3.8 L; *Drosophila*: 21±3.8 L). If anything, this similarity was increased when considering the more unrealistic parameter set 2 that described the experimental data better. Consequently dendritic integration properties affecting synaptic democracy were well conserved. Synaptic democracy in amplitude as expressed in the current transfer between the dendrite root and the rest of the neuron was qualitatively identical between *Calliphora* and *Drosophila* (Figure 3C). Also, temporal synaptic democracy as expressed by the temporal delays between dendrite root or dendrite tip and the rest of the neuron (Figure 3C) was similar but slightly scaled in *Drosophila* because of the difference in the membrane time constant. Again, these similarities were only affected slightly when the alternative set of passive membrane properties was used for the *Drosophila* electrotonic model (Figure 3C, orange).

**Figure 4:**
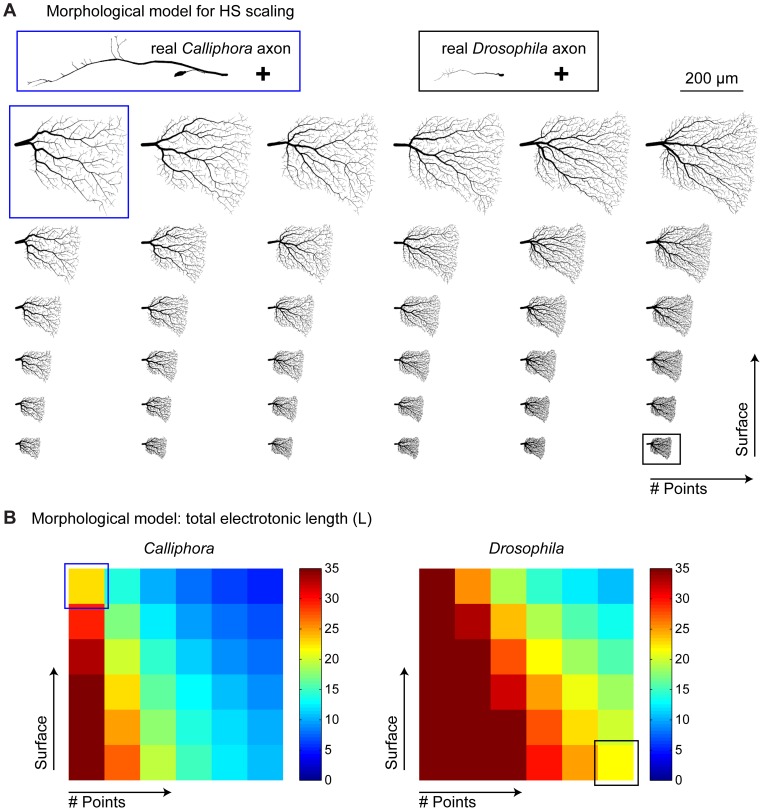
Electrotonic analysis of a morphological model for scaling HS cells. (A) Database of models generated by pairing either a sample *Calliphora* (blue box) or a sample *Drosophila* HSE axon (black box) to a synthetic dendrite obtained from a sample *Calliphora* HSE dendrite contour but scaled in overall size (surface) and in complexity (number of branch points). Upper left (blue box) and lower right (black box) model dendrites correspond to *Calliphora* and *Drosophila* dendrite measures respectively. (B) Corresponding to the morphological model databases in A, total electrotonic length is shown for *Calliphora* (left) and *Drosophila* (right) morphological models. Models with realistic morphologies for *Calliphora* (blue box) and *Drosophila* (black box) are in the same range but scaling surface area or number of branch points changes these measures.

To test how robust these properties were with morphological changes we designed a morphological model with variable branch point numbers and dendrite surface areas. To do this we selected one sample dendrite contour from a Calliphora HSE and generated synthetic morphologies using the method described above but varying both the number of target points and the scaling factor for the surface area. These synthetic dendrites were appended to either a *Calliphora* or a *Drosophila* axon (Figure 4A). Note that the only differences between a *Calliphora* neuron and a *Drosophila* neuron were therefore given by (1) the appended axons, (2) the diameters mapped onto the dendrites and (3) the passive membrane properties. Summed electrotonic length measures for this database of morphological models (Figure 4B) showed that the passive membrane properties are indeed selective for the particular overall morphology. In conclusion however, dendritic integration properties are largely unaffected by the scaling procedure and changes in passive membrane properties are helpful to further stabilise the electrotonic skeleton.

### Integration of visual responses in HS cells

The model can then be used to study the responses to visual stimulation of HS cells in a comparative way between *Calliphora* and *Drosophila*. We focused here on the integration of large-field visual inputs that have been extensively studied in *Calliphora* HS cells [16]. As mentioned above, the dendritic arrangement of HS cells is retinotopic and the Ca^2+^ distribution within the dendrites reproduces the motion image in the visual field [43]. One function of the HS cell dendrite is to integrate democratically the motion vectors present in the individual parts of the visual field and to smooth out irregularities due to textures in the moving background in both space and time [18].

**Figure 5:**
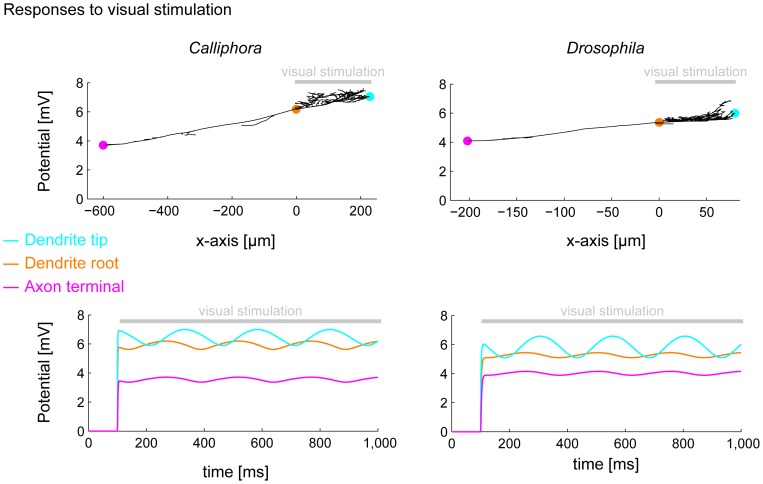
Visual responses in *Drosophila* and *Calliphora* HS cells. *Calliphora* (left) vs. *Drosophila* (right) HSE cell model responses to full field visual stimulation (top) and full field sinusoidal conductance injections in the dendrite with the phase corresponding to the x-axis location of the conduction injection site (bottom). Cyan, orange and pink dots in top panels indicate dendrite tip, dendrite root and axon terminal locations respectively for which voltage time traces are plotted in bottom panels. Grey bars indicate stimulation region (top panels) and time onset of stimulation (bottom panels).

In order to simulate visual responses we distributed a total synaptic conductance in the terminal branches of the dendrites that corresponds to the total input conductance of the cell as derived from visual stimulation recordings in *Calliphora* HS cells [15]. Since the HS cell membrane potential responds to visual stimulation in a graded manner, simulations using passive electrotonic models produced good results. Synaptic conductances of about 9 pS×577 = 5.2 nS were required in the *Drosophila* HS cell model compared to about 900 pS×278 = 250 nS in the *Calliphora* HS cell model (Figure 5, top panels) and achieved voltage responses to large-field visual stimulation of about 5 mV at the electrode location (in the axon or the soma) for both cells. This indicates that the amplitudes of synaptic conductance indeed match the input resistance and therefore that the passive membrane properties of the cell match the synaptic conductance. The voltage distributions throughout the cells were similar; compare in particular the dendritic tip where the synapses were located (Figure 5, cyan dots), the dendrite root where the signals are integrated (Figure 5, orange dots) and the axon tip where signals are conveyed to neurons that descend to the thoracic ganglia involved in flight muscle control (Figure 5, pink dots).

Finally, we studied how dendritic integration averages out modulations in the visual input due to textures in the visual background. We inserted sinusoidally modulated synaptic conductances along the dendrites of the model HS cells reflecting visual inputs due to a moving spatial grating. The phase was proportional to the x-coordinate for each synaptic input and the sinusoidal input covered the dendritic span with exactly one period. In both *Calliphora* and *Drosophila* model cells, the modulations vanished at the level of the dendrite root and the axon tip (Figure 5, bottom panels; see also movie S1). In conclusion, also the visual response properties between *Calliphora* and *Drosophila* HS cells were qualitatively similar throughout the neuron in the electrotonic compartmental models.

## Discussion

In recent years comparison of *Drosophila* and *Calliphora* Lobula Plate circuits have revealed close similarities in anatomical and computational features [24], [25], [44], [45]. We focused on one type of neuron, the HS cell, to study specifically to which extent it is modified to compensate for the extreme differences in size between both species. We showed that *Drosophila* HS cells follow the same branching principles as *Calliphora* HS cells and that the underlying electrotonic architecture is well conserved. We find also that the morphology obeys essentially isometric scaling and that even drastic scaling alterations do not per se challenge dendritic integration features such as synaptic democracy and responses to visual motion. Furthermore, total length and number of branch points, i.e. dendrite complexity, are strongly linked to each other by optimal wiring constraints (Figure 1D) [33].

Even though the general anatomical features were roughly scaled isometrically, two notable features departed from this rule. Firstly, while the average diameter values were scaled isometrically, the distribution of the branching diameters was altered possibly to conserve dendritic synaptic democracy. Secondly, and more surprising, the complexity of *Drosophila*'s HS cells was increased compared to *Calliphora*. The lower spatial resolution of the *Drosophila* visual system with roughly 700 ommatidia per eye [39] vs. 4,500 in *Calliphora* [40] would suggest a lower complexity in the retinotopically organised branching structures of LPTCs since their function is to integrate signals from individual columnar elements over large parts of the visual field.

The morphological model of *Drosophila* cells indicated that the trade-off between cable cost and short conduction delays is in favour of short cables in a similar way as had been the case for *Calliphora* cells [12], [28]. Together with the planar organisation of LPTCs within the Lobula Plate, this finding sets them functionally in one common group with cerebellar Purkinje cells that were suggested to also maximise their connectivity repertoire [11], [46], [47]. The low importance of conduction delays in the morphological model for both types of HS cells, thereby indicating less electrotonic compartmentalisation [10] is highly suggestive of a similar functional role. This function does not seem to be affected by general scale.

The electrotonic properties of the cells indicate that the specific membrane properties did not change very much. Changes in membrane resistivity in the scaling process led to higher input resistances without compromising dendritic integration. As a result however, predicted synaptic currents are much smaller in *Drosophila* than in *Calliphora* HS cells. This could in turn result in smaller metabolic costs generally associated with higher input resistances [48]. The differences in electrotonic properties that were seen are hard to resolve since experimental measurements were performed using sharp electrodes in axons of *Calliphora* HS cells but with patch electrodes in somata of *Drosophila* HS cells. While the former have been shown to introduce higher leak conductances, the latter have unknown influences on ion concentrations [49]. Studies performed in maturing invertebrates also describe the conservation of electrophysiological features in the nervous system even with large differences in size [50], [51]. Interestingly, the functional syntax (e.g. spike timing), but not the absolute response intensity were conserved within growing cricket neurons, supporting the idea that functional concepts rather than the detailed physical sizes of features are encoded genetically [52], [53]. In general the number of detailed electrotonic studies in *Drosophila* is still limited. Antennal lobe projection neurons exhibit different function, morphology and electrotonic properties compared to the HS cells described here [54]. However, input resistance measurements of around 220 MΩ in *Drosophila* VS cells, another class of LPTCs, matches our measurements in HS cells [32].

Most strikingly and in accordance with previous electrophysiological recordings [24], [25], the dendritic integration properties and the simulated responses to visual stimulation were extremely similar in the *Calliphora* and *Drosophila* HS cell models. Despite the anatomical scaling of 60× between the two fly species, the similarity in the electrotonic structure seems to be rather robust. While this requires some adjustments in the set of membrane properties, the range of adequate parameters is rather large. The overall importance of morphology for neural computation has been emphasised in many studies [55]–[57]. Dendrite structure plays a large role for spiking responses [56] and theoretical discussions on preserving synaptic integration through adjustments of morphological and electrotonic scaling properties of neurons have been held [3], [58]. We show here in a combined electrophysiology, morphology and modelling study that iso-electrotonic scaling is feasible with minor adjustments in passive membrane properties and anatomy in the fly HS cell. We have provided evidence that the morphological backbone is important but robust over a wide range of scaling alterations in terms of the implementation of dendritic computations. By dissecting morphological and electrotonic scaling features, we show that neural function and many electrotonic properties are not compromised by scaling. We therefore conclude that a conservative scaling as proposed previously is comparably simple to achieve.

## Supporting Information

Figure S1 ***Drosophila*** **HS morphology database.** HSN and HSE cells were genetically tagged with GFP and all HSN and HSE cells from five flies were imaged with confocal microscopy (left two columns) and reconstructed (right two columns; HSN – red, HSE – black). The two columns each represent the left and right lobula plate of the same animal so that each row corresponds to the data obtained from one animal.
(TIF)

Movie S1 **Responses of** ***Drosophila*** **and** ***Calliphora*** **HS model cells to a moving sinusoidal grating.** The dendrite receives sinusoidally modulated inputs where the phase depends on the x-coordinate and the pattern covers the dendrite with exactly one period of the sinusoidal input. The y-axis shows the local membrane potential instead of the correct HS cell y-coordinate (see Figure 5 for more details).
(AVI)
