# Supplementary figures and images for: Preserving Neural Function under Extreme Scaling
Source: PLoS One. 2013 Aug 19;8(8):e71540. doi: 10.1371/journal.pone.0071540 (PMC3747245; doi:10.1371/journal.pone.0071540)

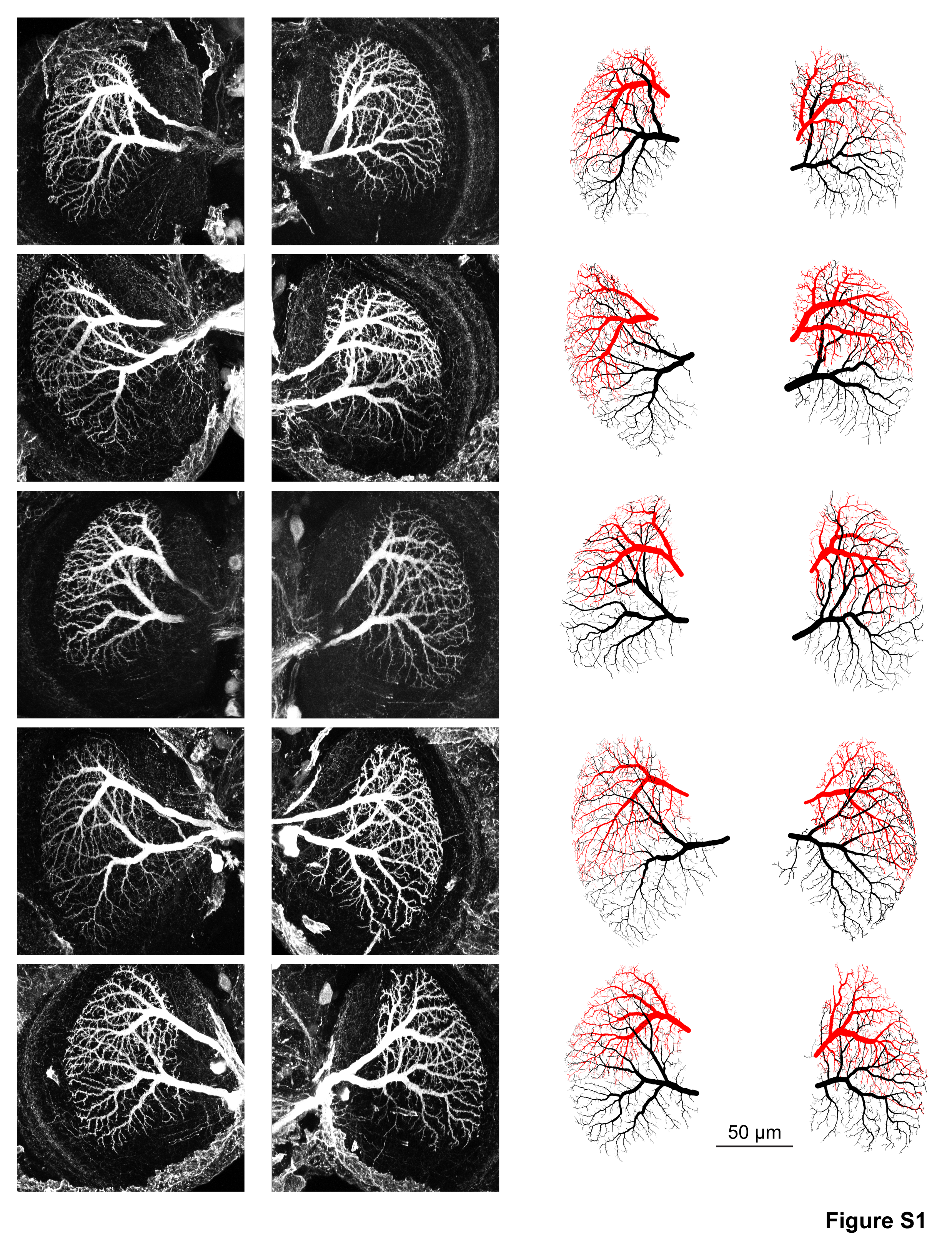

Supplement: Figure S1 — Drosophila HS morphology database. HSN and HSE cells were genetically tagged with GFP and all HSN and HSE cells from five flies were imaged with confocal microscopy (left two columns) and reconstructed (right two columns; HSN – red, HSE – black). The two columns each represent the left and right lobula plate of the same animal so that each row corresponds to the data obtained from one animal. (TIF) [file pone.0071540.s001.tif]
